# Role of the striatum in counterfactual information seeking

**DOI:** 10.1093/scan/nsag012

**Published:** 2026-03-10

**Authors:** Johnny King L Lau, Michiko Sakaki, Lily FitzGibbon, Jasmine A L Raw, Kou Murayama

**Affiliations:** School of Psychology and Clinical Language Sciences, University of Reading, Reading, RG6 6ET, UK; Hector Research Institute of Education Sciences and Psychology, University of Tübingen, Tübingen, 72072, Germany; Research Institute, Kochi University of Technology, Kami, 782-8502, Japan; Division of Psychology, University of Stirling, Stirling, FK9 4LA, UK; School of Psychology and Clinical Language Sciences, University of Reading, Reading, RG6 6ET, UK; Hector Research Institute of Education Sciences and Psychology, University of Tübingen, Tübingen, 72072, Germany; Research Institute, Kochi University of Technology, Kami, 782-8502, Japan

**Keywords:** regret, reward, dopamine, information seeking, decision making, counterfactual thinking, uncertainty

## Abstract

After making a choice, we sometimes seek information to explore alternative realities (i.e. the outcome of an option that we have not chosen). Recent research suggests that people have a strong urge to seek such counterfactual information (counterfactual curiosity), even though it can lead to negative emotions, such as regret. In the present study, we used an adapted Balloon Analogue Risk Task with functional magnetic resonance imaging to investigate brain regions associated with counterfactual curiosity. We replicated previous behavioral findings that people are willing to find out counterfactual information (how much more they could have won) after winning their game. We also observed the emotional cost associated with counterfactual curiosity, such that participants felt stronger negative emotions after seeking counterfactual information. Brain imaging results revealed that the choice to seek counterfactual information is associated with stronger activity in the caudate, brain regions implicated in processing of intrinsic and extrinsic rewards. In addition, the caudate and the nucleus accumbens showed greater activity when participants realized that they could have won more points. These results suggest that the striatum plays a crucial role in seeking and processing counterfactual information.

## Introduction

Curiosity is a fundamental motivation that enables us to seek and learn new information even when there are no clear extrinsic rewards associated with the information (Kashdan and Silvia 2009). It plays a key role in supporting academic achievement (Von Stumm *et al.* 2011), well-being (Lydon-Staley *et al.* 2020), and adaptive aging (Sakaki *et al.* 2018). However, seeking information out of curiosity is not always adaptive; people sometimes experience a strong motivational urge to seek information even if it leads to negative emotional consequences (Hsee and Ruan 2016, Oosterwijk 2017, Lau *et al.* 2020, Yagi *et al.* 2023, Dezza *et al.* 2024). Building on these observations, there is a growing agreement that curiosity has incentive salience and induces strong motivational urges (FitzGibbon *et al.* 2020, Litman 2010) as observed with external rewards, such as food and money (Berridge *et al.* 2009).

Emotional costs incurred by curiosity are particularly pronounced in situations where people experience *counterfactual curiosity—*curiosity for counterfactual information (i.e. alternatives to reality). For example, after buying a new house in a nice area, one may be curious about the prices of other properties in the same area, frequently checking property websites, and wondering if they should have waited for a cheaper option. Such counterfactual comparisons are known to induce strong negative emotions—in particular regret (Loomes and Sugden 1982). Despite the emotional cost, humans and other animals are often willing to explore counterfactual information and face emotional consequences afterwards (FitzGibbon *et al.* 2021, 2019, Wang and Hayden 2021, Bogani *et al.* 2025). These results suggest that counterfactual information has strong incentive salience (FitzGibbon and Murayama 2022).

Previous neuroimaging studies demonstrated that curiosity is associated with the dopaminergic system in the brain, including the striatum, the substantia nigra (SN), and the ventral tegmental area (VTA; Kang *et al.* 2009, Gruber *et al.* 2014, Gruber and Ranganath 2019, Lau *et al.* 2020, Oosterwijk *et al.* 2020)—brain regions typically associated with incentive salience of external rewards (Adcock *et al.* 2006, Berridge *et al.* 2009, Lawrence *et al.* 2012). For example, Lau *et al.* (2020) found that people are willing to play a gambling game to satisfy their curiosity (by seeking information) or hunger (by obtaining food), even though the decision involved the risk of receiving electric shocks; greater activity in the caudate during the choice was also predictive of greater tendencies to accept the gamble to satisfy their curiosity or hunger. These findings suggest that the dopaminergic system plays a key role in impulsive behavior out of incentive salience generated by non-instrumental information as seen in external rewards.

**Figure 1:**
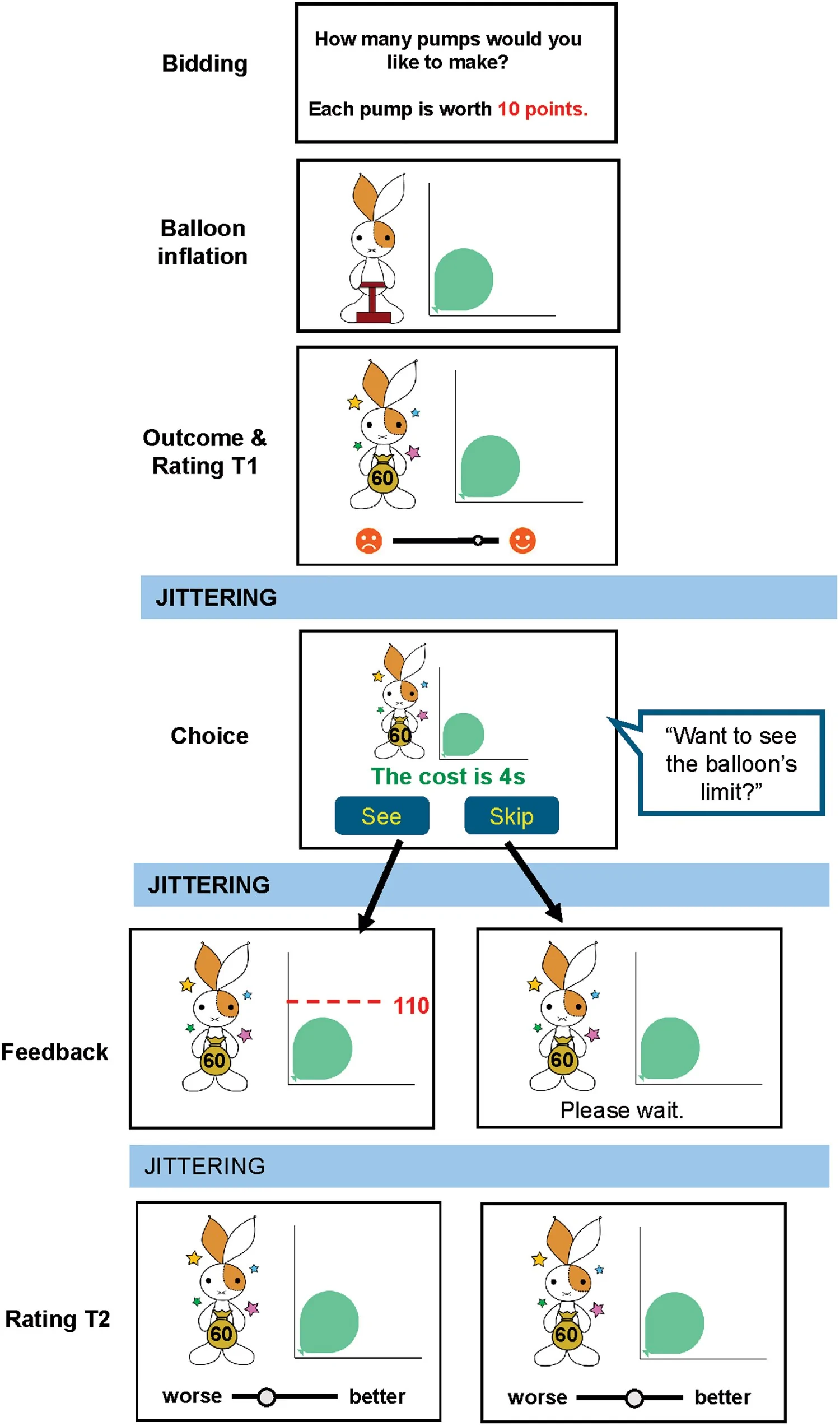
Schematic representation for a bank trial where participants chose to see the counterfactual information (left) or not (right). The task was developed based on FitzGibbon et al. (2021).

In the present study, we addressed the role of the dopaminergic system in counterfactual curiosity using a paradigm known to induce counterfactual curiosity and regret (FitzGibbon *et al.* 2021). Participants played a modified version of the Balloon Analogue Risk Task (BART; Lejuez *et al.* 2002) in an MRI scanner. On each trial (Fig. 1), they first pumped up a balloon and then found whether the balloon remained intact (‘bank’) or exploded (‘bust’) in an outcome phase. The larger the balloon was inflated, the more points they were able to collect as long as the balloon remained intact; however, when the size of the balloon exceeded a limit, which was randomly determined across trials, the balloon exploded, and participants were not able to earn any points. After this outcome phase, participants were asked whether they would want to seek information about the balloon’s limit for the trial (‘the choice phase’). When they indicated that they would want to seek the information, they were next shown how much they could have earned on the trial (‘the feedback phase’). Given that the limit of the balloon was randomly determined for each trial, the information about the balloon’s limit did not have any utility value for future performance. Furthermore, when participants sought the information about the balloon’s limit in the bank trials (where the balloon remained intact because it did not exceed the limit), they almost always found that they could have earned more points; thus, counterfactual information in the bank trials had a high likelihood to lead to negative emotions. In summary, the design is ideal to examine counterfactual curiosity for non-instrumental information that induces regret. Using this paradigm, previous research found that people are willing to seek information about the balloon’s limit, even though it induces negative emotions afterwards (FitzGibbon *et al.* 2021). The present study aimed to examine the roles of the dopaminergic system in one’s decisions to seek counterfactual information despite its emotional costs.

Given our focus on counterfactual information seeking and resulting regret, our behavioral and brain imaging analyses primarily focused on the bank trials (trials where participants successfully earned points) as done in previous research (FitzGibbon *et al.* 2021). In our behavioral analysis, we expected to replicate previous findings that seeking counterfactual information would induce negative emotions in the bank trials. We also expected that the effects of counterfactual information on negative emotions would be greater when participants realized that they could have won larger than smaller quantities of additional points (i.e. when the size of the missed opportunity is larger than smaller) as observed in FitzGibbon *et al.* (2021). In addition, we examined whether participants adjusted their behavior based on the counterfactual information (i.e. missed opportunities) even though the information did not have any instrumental value.

In our brain imaging analyses, we first examined brain activity during the choice phase. Building on previous findings on curiosity and information seeking described above (Lau *et al.* 2020), we expected that the dopaminergic system, particularly the caudate, shows greater activity when people decide to seek counterfactual information than when they decide not to in the bank trials. We next examined brain responses to the counterfactual information by analyzing them during the feedback phase. Previous research revealed that the striatum changes its activity when people realize that they have missed large opportunities and experience regret (Büchel *et al.* 2011). A recent meta-analysis also showed that regret is associated with stronger levels of activity in the nucleus accumbens (NAcc; Varma *et al.* 2023). Thus, we expected greater levels of activity in the striatum when participants received the counterfactual information than when they did not. The activity level was also expected to reflect the size of the missed opportunity, given that larger missed opportunities were expected to be associated with stronger negative emotions as described earlier.

## Materials and methods

### Participants

Forty-one participants (*M*_age_ = 21.22, SD = 3.75; 13 males and 28 females), recruited from the University of Reading (37 undergraduates, two master students, and two PhD students), took part in the study. Participants provided written consent based on the protocol that was given a favorable opinion by the University of Reading’s Research Ethics Committee. Data from three participants were excluded prior to data analyses; one participant did not provide any emotion ratings, and the other two participants exhibited excessive motion in their fMRI scans (>3 mm displacement between adjacent volumes in any motion direction; see FMRI Preprocessing for more details). This resulted in 38 participants (*M*_age_ = 21.42, SD = 3.82; 12 males and 26 females); among them, twenty-eight identified themselves as White, five as mixed, three as Asian, one as African, and the remaining one participant as British. One participant always sought information in the bank trials. As such, data from this participant could not be included in analyses when the corresponding condition (i.e. choices not to seek information in the bank trials) was included. This means that across analyses, the number of participants included varied between 37 and 38.

### Design and behavioral procedure

Following the procedure adopted by FitzGibbon *et al.* (2021), we employed their modified version of the BART in this experiment. Each trial (Fig. 1) began with a bidding phase, during which participants were presented with the number of points each pump was worth in an upcoming trial (‘the pump value’; randomly sampled between 1 and 100); they were asked to indicate how many times they wished to pump the balloon in that trial by selecting a value between 0 and 12 pumps within a 4-second window (‘the number of pumps’). The bidding phase was followed by an animation illustrating a cartoon rabbit inflating the balloon according to the number of pumps indicated by the participant. With each pump, the balloon visibly expanded. Participants were next shown whether the balloon remained intact (outcome = ‘bank’) or burst (outcome = ‘bust’). During this outcome phase, participants were asked to indicate their emotional states by moving the marker along a visual analogue scale with pictures of a sad face on the left and a happy face on the right (within 3 seconds; Time 1). This emotional rating was followed by a jittered interval (lasting 2 to 9 seconds) and a choice phase, during which participants were shown how much extra time (ranging between 0 and 6 seconds) it would cost them to wait at the end of that trial if they decided to see the balloon’s limit; participants had to indicate whether they would want to see the limit or not within 2.5 seconds. After this choice phase, another jittered interval (lasting 2–9 seconds) preceded the feedback phase (lasting 2 seconds). As feedback, participants were shown the balloon’s maximum capacity when they had chosen to see it in that trial; otherwise, they saw the same page displayed during the choice phase. Following another jittered interval (2–9 seconds), participants rated how their emotional state had changed on a visual analogue scale with the left anchor as ‘worse’ and the right anchor as ‘better’ (Time 2). After the rating, if participants chose to see the feedback, they had to wait for the duration indicated during the choice phase (0–6 seconds). Finally, a jittered fixation period (1–3 seconds) preceded the start of a new trial.

Participants completed two runs, each of which included 37-40 trials. Some trials took longer than others due to participants’ choice to seek the counterfactual information and the associated waiting time. We set a maximum time for the scan as well as the maximum number of trials; each run ended whichever came first. Participants were compensated for their time in the study but did not receive an additional bonus based on their performance.

### MRI data acquisition

Whole-brain functional and anatomical images were acquired using a 3.0 Tesla Siemens Magnetom scanner with a 32-channel head matrix coil at the Center for Integrative Neuroscience and Neurodynamics (CINN), University of Reading. Functional images were acquired using a T2*-weighted gradient-echo echo planar imaging pulse sequence with 37 axial slices (in-plane resolution of 3 × 3 × 3 mm), interleaved from bottom to top (echo time (TE): 30 ms; repetition time (TR): 2000 ms; flip angle: 90°; field of view (FOV): 1344 × 1344 mm^2^; in-plane matrix: 64 × 64). A high-resolution T1-weighted three-dimensional anatomical image was also collected, using an MPRAGE-gradient sequence with 176 × 1-mm slices (in-plane resolution of 1 × 1 × 1 mm; TE: 2.29 ms; TR: 2300 ms; inversion time (TI): 900 ms; FOV: 250 × 250; flip angle: 8°).

### Behavioral analysis

Emotional ratings at Time 1 and Time 2 were scaled into a range between -10 and 10 prior to analyses. Participants failed to indicate the number of pumps within the time window in some trials (the mean proportion of those trials = .008). We excluded these trials from our analyses. Behavioral analyses were performed with R version 4.1.2 (R Core Team 2021). Figures were generated by the ‘ggplot2’ package (Wickham *et al.* 2016). Comparisons across two conditions (e.g. the bank trials vs. the bust trials or when information was sought vs. not) were performed with the ‘afex’ package (Singmann *et al.* 2015).

We also ran a series of trial-level analyses with the ‘lme4’ package (Bates 2010). In these trial-level analyses, all independent variables were person-mean centered prior to the analysis; we also included random intercepts of participants and random slopes of within-person predictors across participants (unless otherwise indicated). First, we examined the effects of pump value on the number of pumps participants indicated in the bidding phase. Second, we used a generalized mixed-effects model with the *glmer* command to examine factors that affected participants’ choice to seek counterfactual information. The dependent variable was the choice to seek counterfactual information; independent variables were (a) the number of pumps participants indicated, (b) the pump value, and (c) the time cost required for counterfactual information.

Next, the within-person effects of the missed opportunities (how much more participants could have won on each trial) on emotions were examined; the dependent variable was emotional ratings at Time 2, whereas the independent variables were (a) the missed opportunity, (b) the number of pumps participants indicated, (c) the choice (seek information vs. not), (d) the interaction between the missed opportunity and the choice, (e) the interaction between the number of pumps and the choice, and (f) emotional ratings at Time 1. The size of the missed opportunities was quantified by a difference between the number of pumps participants indicated and the limit of the balloon for each trial.

We also investigated whether participants’ subsequent behavior was affected by the counterfactual information. The analysis focused on subsequent trials after participants sought counterfactual information in bank trials. The dependent variable was the number of pumps participants indicated; independent variables were the number of pumps they indicated in the previous trial, the missed opportunity in the previous trial, emotional ratings (at Time 2) in the previous trial, and an interaction between missed opportunity and emotional ratings.

### FMRI preprocessing

Functional MRI (fMRI) data were preprocessed with fmriPrep (Esteban *et al.* 2019). Resulting data were then spatially smoothed using a 9-mm Gaussian kernel with the Statistical Parametric Mapping 12 (SPM12; https://www.fil.ion.ucl.ac.uk/spm/). Using SPM12, we also performed spatial realignment of the echo-planar imaging (EPI) volumes to correct for movement artifacts, and this step estimated motion parameters of each volume. With this step, we identified scans that showed abrupt movements (> 3 mm displacement between adjacent volumes in any motion direction).

### Regions of interest (ROI)

We conducted ROI analyses, focusing on the regions implicated in curiosity and regret (Lau *et al.* 2020, Varma *et al.* 2023): the bilateral NAcc, the bilateral caudate, and the substantia nigra/VTA (SN/VTA). The SN/VTA masks were provided by Jessica Mollick, drawn based on Eapen *et al.* (2011). The bilateral NAcc and caudate masks were created based on the Harvard-Oxford Subcortical Probability Atlas with a probability of 80%. We *a priori* set a relatively stringent threshold (80%) to reduce the chance of including other regions in the masks.

### FMRI analysis

Image analysis was carried out using FSL FEAT (fMRI Expert Analysis Tool, https://fsl.fmrib.ox.ac.uk/fsl/fslwiki/FSL). For each run, the BOLD responses were modeled with a general linear model (GLM) with the following events: (a) the outcome phase, (b) the choice phase when participants decided to seek the counterfactual information, (c) the choice phase when they decided not to seek the counterfactual information, (d) the feedback phase when they found the balloon’s limit (due to their choice in the choice phase), and (e) the feedback phase when they did not see the balloon’s limit (again due to their choice); for all of these events, we modeled the bank vs. bust trials separately. We also included additional regressors for emotion ratings at T1 and T2 (separately), waiting times, and the bidding phase. To examine the brain regions relevant to counterfactual curiosity, we contrasted BOLD signals in the choice phase when participants decided to seek information in the bank trials vs. the choice phase when participants decided not to seek information in the bank trials. To identify brain regions relevant to regret due to counterfactual information, we used a contrast between the feedback phase when participants sought information in the bank trials vs. the feedback phase when they did not seek information in the bank trials.

The outputs from the first-level analysis were merged across runs for each participant using a fixed-effects analysis in FSL’s FEAT. Beta values were then extracted using the FSL’s featquery command for each contrast of interest for each participant in each ROI. For the NAcc and caudate, the beta values for the left and right hemispheres were averaged for each participant. They were then analyzed with R version 4.1.2 (R Core Team 2021) to examine if each area shows greater activity in one condition over the other condition. We employed false discovery rate (FDR) corrections for multiple comparisons across ROIs. To visualize the activity patterns, we used the ‘ggplot2’ package (Wickham *et al.* 2016).

This main analysis was followed by a series of additional analyses. First, we performed an exploratory whole-brain analysis to examine how the choice to seek information affects BOLD signals beyond our ROIs during the choice and feedback phases (see Supplementary Materials). Second, we conducted a seires of parametreic modulation analyses; in these analyses, the first-level analysis was carried out again with another GLM that included all the regressors included in the main analysis as well as additional parametric modulators (these parameters were mean-centered for each run for each participant).

#### Effects of missed opportunity

To investigate whether the missed opportunity affected brain activity during the feedback phase, the first level analysis was carried out with another GLM with two parametric modulators based on the missed opportunity when participants sought information (one for the bank trials and the other for the bust trials). The missed opportunity was included as a parameter to modulate the brain activity to the feedback phase in the GLM model.

#### Downstream effects of counterfactual information

To examine if brain activity induced by counterfactual information predicts subsequent behavior, we ran an additional analysis, which included a parametric modulator based on a change in the number of pumps between the current and a subsequent trial. This parameter was included as a parameter to modulate the brain activity to the feedback phase in the GLM model. A similar analysis was also conducted with the absolute value of the change in the number of pumps between the current and a subsequent trial.

#### Effects of pump value and cost

Two additional GLMs were performed to examine if the pump value and time cost were associated with activity in our ROIs during the choice phase. In an analysis on the effects of pump value, we added two additional parametric modulators based on the pump values for the choice phase: one for the bank trials and another for the bust trials. In an analysis on the effects of cost, these parametric modulators were replaced by those based on the time cost values: one for the bank trials and another for the bust trials. In both cases, parametric modulators were included as a parameter to modulate the brain activity to the choice phase in the GLM model.

## Results

As described earlier, given that our primary research questions concern counterfactual curiosity and resulting negative emotions, our main analyses focused on bank trials. The results from the bust trials are reported in the Supplementary Materials.

### Behavioral results

#### Balloon size indicated in the bidding phase

**Figure 2:**
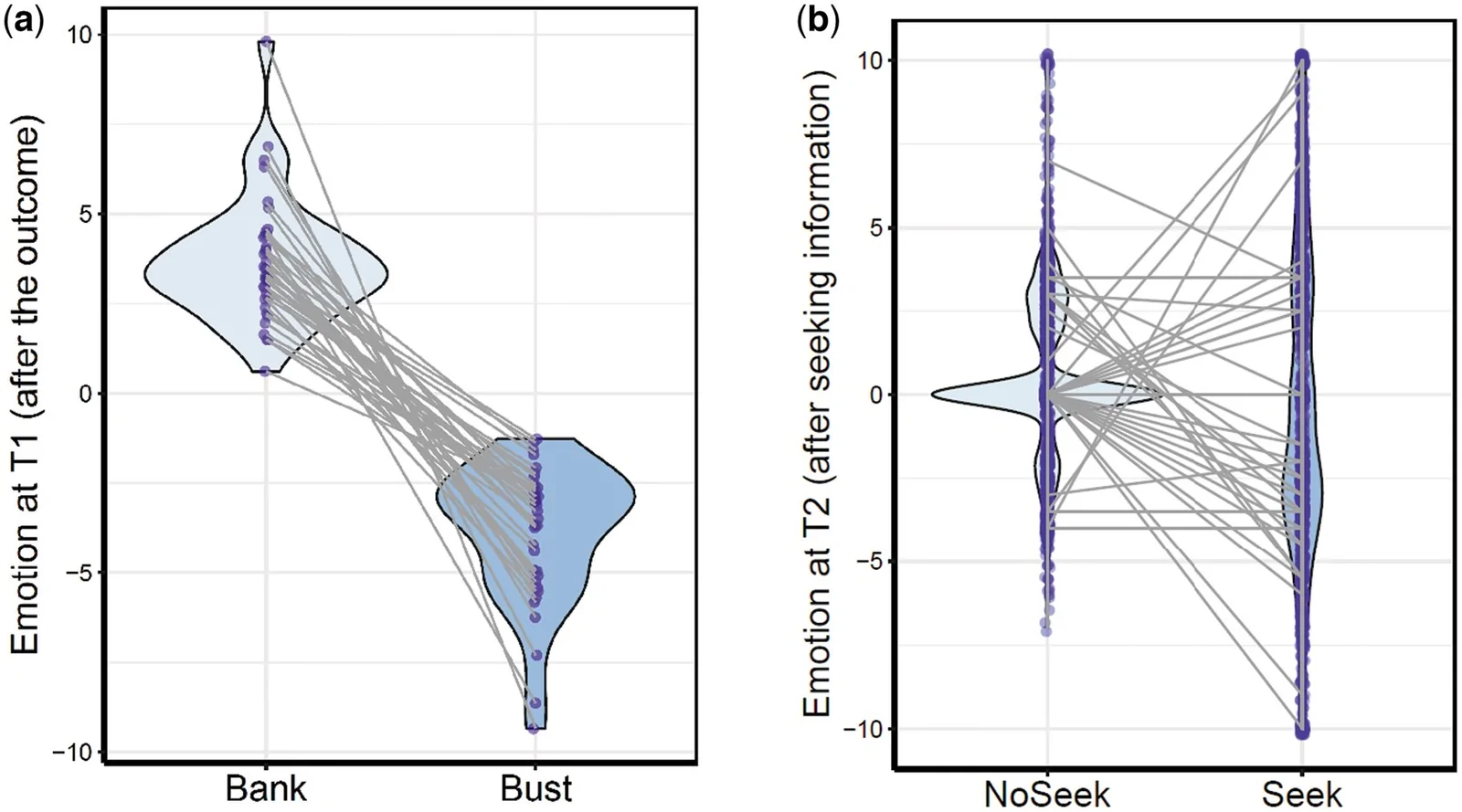
(a) Effects of outcomes on emotion ratings at T1 (higher values mean more positive emotions; each line represents each participant). (b) Effects of choice on positive emotional ratings at Time 2 (each line represents each participant).

Participants pumped more when the pump value was smaller, *t*(2901) = −12.20, *P* < .001 (Table S1 in Online supplementary material), and the balloon did not explode in a previous trial (Table S2 in Online supplementary material), *t*(30.21) = −3.572, *P* < .001. On average, they pumped the balloon 5.13 times (SD = 1.06) out of 12 across trials, which was lower than the average limit of balloons (*M *= 6.10, SD = 0.40). Thus, participants were relatively conservative, as observed in a previous study (FitzGibbon *et al.* 2021). This conservative choice resulted in a larger number of bank trials (*M* = 0.62, SD = 0.08) than bust trials (*M* = 0.38, SD = 0.08). Participants reported significantly more positive emotions after the outcome phase (at Time 1) in the bank trials (*M *= 3.67, SD = 1.71) than the bust trials (*M *= −3.88, SD = 1.90; Fig. 2a), *F*(1, 37) = 199.40, η^2^_G_ = 0.817, *P* < .001.

#### Counterfactual information seeking

Participants sought information for the balloon’s limit significantly more in the bank trials (*M* = 0.52, SD = 0.26) than the bust trials (*M* = 0.40, SD = 0.24; Figure S1—see online supplementary material for a colour version of this figure), *F*(1, 37) = 17.51, η^2^_G_ = 0.060, *P* < .001. Among the bank trials, participants sought information significantly more when the pump value was higher, *z *= 2.848, *P* = .004 and the time cost was shorter, *z *= −4.744, *P* < .001 (Table 1). However, the number of pumps indicated in the bidding phase was not significantly associated with the choice to seek counterfactual information (*P* = .662; Table 1).

**Table 1:** Effects of pump value, time costs and the number of pumps on counterfactual information seeking in the bank trials.

Predictors	Estimates	SE	*Z*	*P*
(Intercept)	−0.0473	0.3118	−0.152	.8794
Pump value	0.0064	0.0022	2.848	.0044
Time cost	−1.1484	0.2421	−4.744	<.001
Number of pumps	−0.0151	0.0346	−0.437	.6621
Random effects				
σ^2^	3.29			
τ_00participant_	3.40			
τ_11participant.cost_	1.84			
ρ_01_	0.53			
ICC	0.66			
*N*_participant_	38			
Observations	1789			

*Note:* Random slopes for the number of pumps and pump value were removed due to the convergence error.

#### Downstream effects of counterfactual information

**Figure 3:**
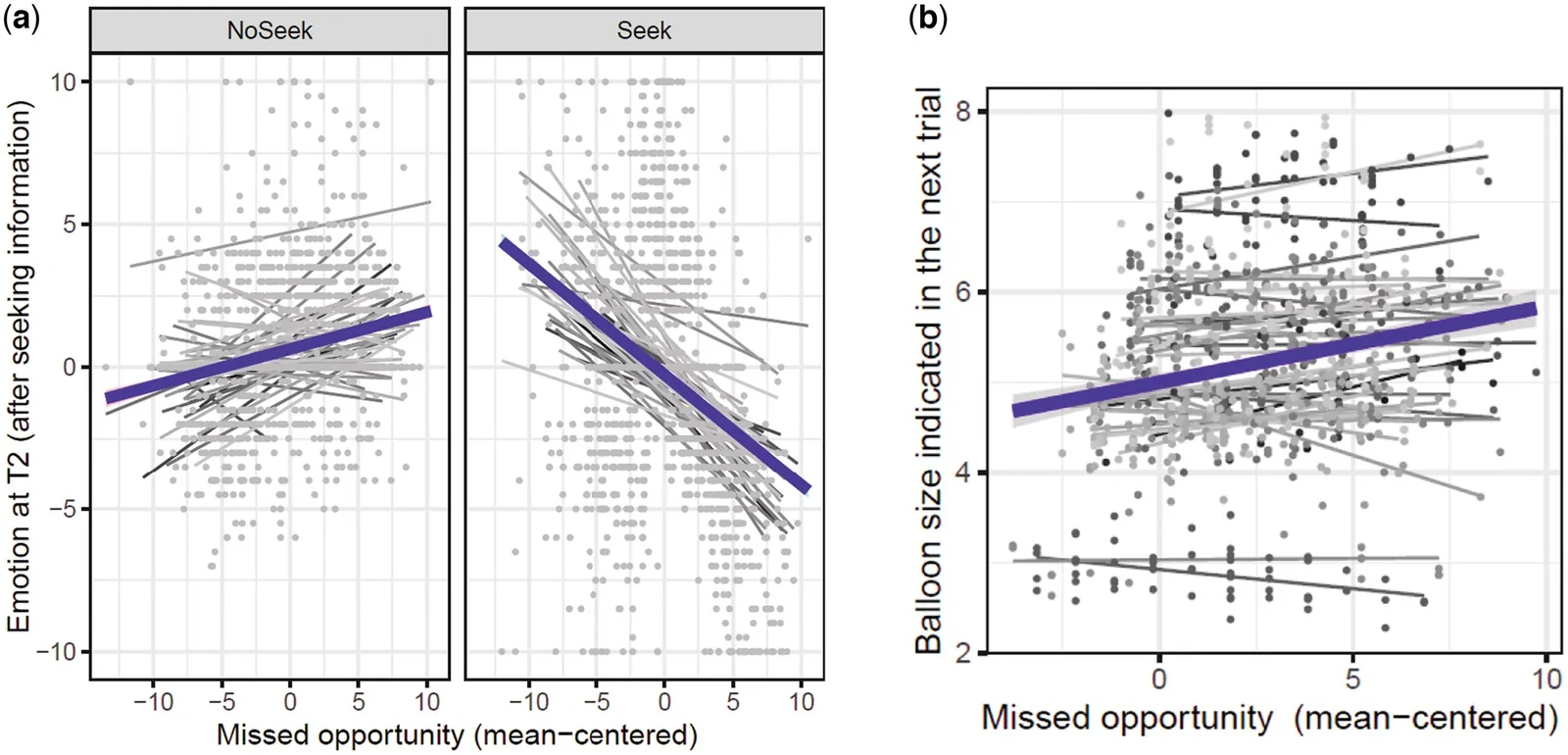
(a) The effects of missed opportunities on emotion ratings at T2 differed depending on whether or not participants sought counterfactual information. (b) The effects of missed opportunities on subsequent bidding behavior (each line represents each participant).

We next examined the effects of counterfactual information on emotions after the feedback phase in the bank trials. Replicating previous findings (FitzGibbon *et al.* 2021), participants reported significantly stronger negative emotions at Time 2 after they sought information (*M *= −0.42, SD = 1.56) than after they did not seek information (*M *= 0.47, SD = 1.39; Fig. 2b), *F*(1, 37) = 13.63, η^2^_G_ = 0.086, *P* < .001. There was also a significant interaction between the choice and the missed opportunities (Table 2), *t*(2744) = −15.18, *P* < .001. Specifically, when participants sought the counterfactual information, they reported more negative emotions at Time 2 when the missed opportunity was larger (Fig. 3a), *t*(46.50) = −11.85, *P* < .001. In contrast, when participants did not seek information, there were no significant effects of missed opportunities (*P* = .70). This makes sense because participants were unable to see the amount of missed opportunities when they did not seek information. We also found that seeking counterfactual information changes subsequent behavior in indicating the number of pumps. Specifically, participants made more pumps after finding a larger missed opportunity, *t*(38.46) = 2.371, *P* = .02 (Table 3 and Fig. 3b). Neither the main effects of emotional responses in a previous trial (Time 2), nor their interaction with missed opportunity was significant (Table 3).

**Table 2:** Effects of counterfactual information seeking on emotions

Predictors	Estimates	*t*	*P*
Intercept	0.58	2.848	.004
Choice (Seek = 1; No-seek = −1)	−0.96	−3.765	<.001
Missed opportunity	−0.17	−3.665	<.001
Number of pumps	0.00	0.058	.954
Emotion at T1	0.39	21.33	<.001
Choice × Missed opportunity	−0.51	−15.18	<.001
Choice × Number of pumps	−0.11	−1.715	.087
Random effects			
σ^2^	8.85		
τ_00participant_	1.33		
τ_11participant.misopp_	0.06		
τ_11participant.c_choice_cent1_	1.78		
ρ_01_	−0.20		
ρ_02_	−0.30		
ρ_12_	−0.08		
ICC	0.16		
*N*_participant_	38		

*Note:* Random slopes for emotion and interactions were not included due to the convergence error.

**Table 3:** Effects of counterfactual information seeking on participants’ choice for the number of pumps in the following trial

Predictors	Estimates	SE	df	*t*	*P*
(Intercept)	5.093	0.1963	44	25.941	<.001
Number of pumps in t-1	0.174	0.0391	880.5	4.442	.00001
Missed opportunity in t-1	0.104	0.0438	38.46	2.371	.0228
Emotion at T2 in t-1	0.024	0.0229	492.9	1.048	.2953
Missed opportunity * Emotion in t-1	0.002	0.0061	369.8	0.304	.7612
Random effects					
σ^2^	3.71				
τ_00_	0.85 _participant_				
τ_11_	0.005 _participant.missed_				
ρ_01_	0.40 _participant_				
ICC	0.22				
*N*	38 _participant_				
Observations	911				

*Note:* Random slopes for number of pumps, emotion and the interaction term were not included due to the convergence error.

### FMRI analysis during choice phase

**Figure 4:**
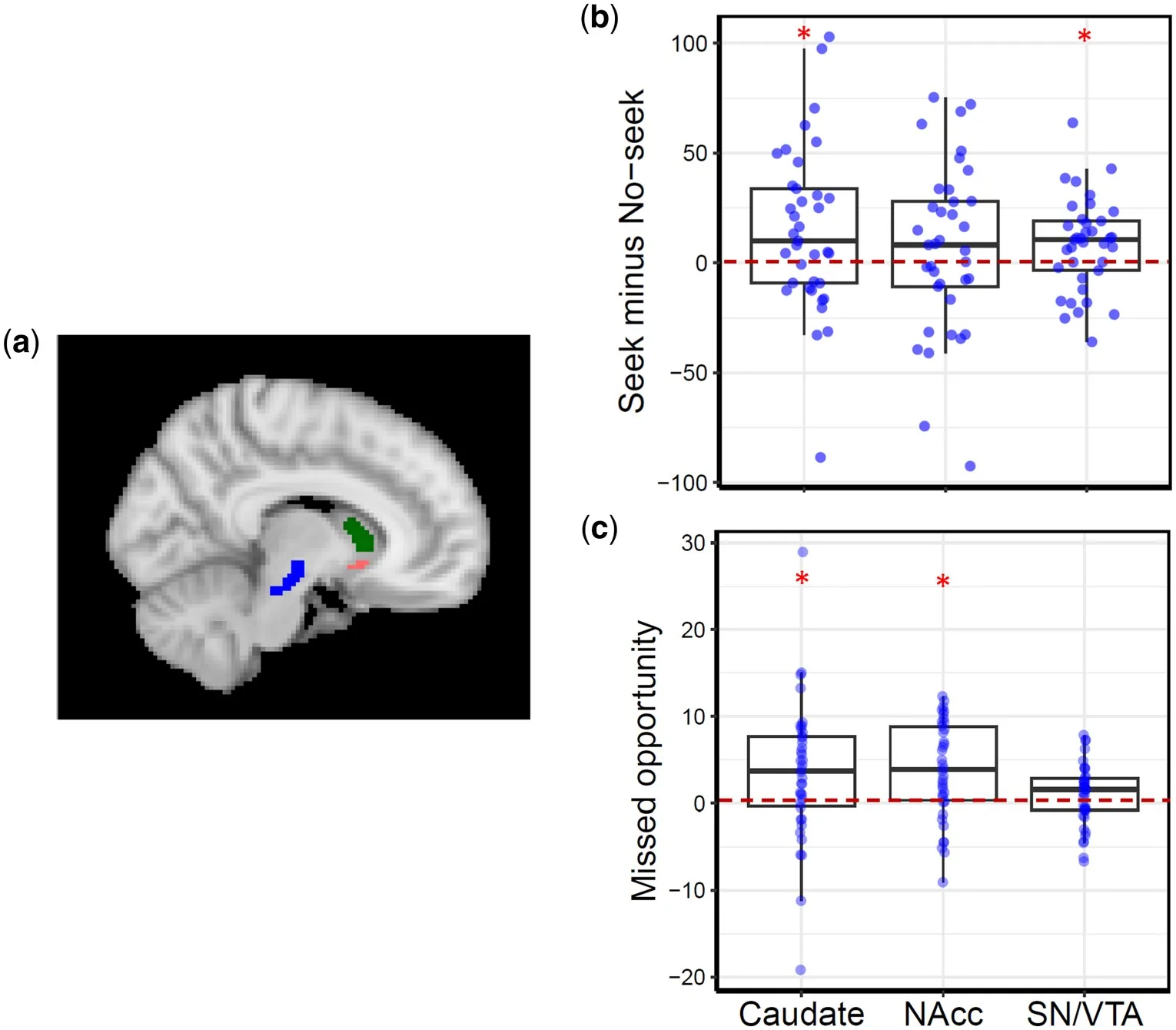
(a) Our regions of interests (blue = SN/VTA, green = caudate and pink = NAcc). (b-c) Results of our ROI analyses. Beta coefficients to show (b) differences between trials where participants sought information vs. not during the choice phase and, (c) the effects of missed opportunity during the feedback phase. Each dot represents each participant. *: p < .05 (FDR).

The caudate and SN/VTA (Fig. 4a) showed significantly greater BOLD signals when participants decided to seek information than when they decided not to (Fig. 4b), *t*s(36) = 2.47, 2.35, *p*s = 0.037, 0.037 (FDR), and *d* = 0.41, 0.39, for the caudate and SN/VTA, respectively. In contrast, the NAcc activity did not show significant differences, *t*(36) = 1.03, *P* = .31 (FDR). Similar results were obtained when we controlled for the effects of pump value or time costs (see Figure S3—See online supplementary material for a colour version of this figure). Together, the results are in line with Lau *et al.* (2020) and suggest that the caudate is relevant to seeking information even due to counterfactual curiosity.

### FMRI analysis during the feedback phase

None of our ROIs showed significant differences when we compared brain activities when participants chose to seek information vs. not during the feedback phase, *t*s(36) = 2.37, 1.23, 1.62, *P*s = .069, .203, .170 (FDR), for the SN/VTA, NAcc, and caudate, respectively. However, our subsequent analysis with parametric modulation revealed that larger missed opportunities were associated with greater BOLD signals in the NAcc and the caudate when participants found the limit of the balloon during the feedback phase, *ts*(37) = 4.21, 2.68; ds = 0.68, 0.43; *Ps* < .05 (FDR) for the NAcc and the caudate, respectively (Fig. 4c). In contrast, the SN/VTA did not show significant effects for the missed opportunities, *t*(37) = 1.662, *P* =.10 (FDR). None of our ROIs were significantly predictive of changes in the number of pumps participants indicated in a subsequent trial (Figure S5—See online supplementary material for a colour version of this figure).

## Discussion

Humans have an incredible ability to reflect on outcomes of our own decisions and to explore alternative realities (i.e. the outcome of an option that we have not chosen). Previous research demonstrated that people spontaneously engage in counterfactual information seeking, exploring what could have happened (FitzGibbon *et al.* 2021, Bogani *et al.* 2025). A similar behavior was also observed for monkeys (Wang and Hayden 2019). In the present study, we replicated these behavioral findings and revealed that people are willing to seek counterfactual information even though it leads to negative emotions. These results are consistent with a theoretical notion that curiosity triggers a motivational urge as seen by external rewards, such as food or money (Marvin *et al.* 2020, FitzGibbon and Murayama 2022, Blain *et al.* 2023).

Our neuroimaging analyses showed that counterfactual information seeking is associated with the dopaminergic reward region, including the caudate and SN/VTA. In the present study, counterfactual information seeking was affected by its cost and value, both of which have been associated with the striatum and SN/VTA (Berridge *et al.* 2009, Westbrook *et al.* 2021). However, the caudate and SN/VTA showed greater levels of activity when people sought counterfactual information than not, even after controlling for the effects of pump value and cost. A growing body of neuroimaging research pointed out that curiosity is supported by the dopaminergic reward systems that play an important role in representing the motivational salience of external rewards (Kang *et al.* 2009, Gruber *et al.* 2014, Lau *et al.* 2020). Our results extend prior findings and suggest that the same brain regions are also important for seeking information to satisfy counterfactual curiosity.

Previous studies revealed that people exhibit greater curiosity for and seek positive information than negative information (Marvin and Shohamy 2016, Sharot and Sunstein 2020). In contrast, counterfactual curiosity has a unique feature: revealing the information and satisfying curiosity leads to negative emotional states. In fact, in the present study, participants felt more negative after seeking information about the balloon’s limit than not seeking the information at all. Counterfactual information seeking was also observed more frequently in the bank trials (i.e. when the information had a high likelihood to lead to negative emotions) than the bust trials (i.e. when the information should not lead to negative emotions), replicating previous findings that it occurs particularly when the counterfactual outcome is likely better than the reality (FitzGibbon *et al.* 2021, Bogani *et al.* 2025). In addition, the striatum and SN/VTA were associated with counterfactual information seeking in bank trials but not in bust trials (Figure S6—See online supplementary material for a colour version of this figure). These results suggest that positive valence does not always enhance curiosity (see also Yagi *et al.* 2023) and that the dopaminergic reward system is crucial for curiosity even when it leads to negative emotions.

Another interesting result concerns the role of uncertainty in counterfactual curiosity. In the present study, participants had to select a balloon size between 0 and 12 pumps; this means that in bank trials where the balloon did not explode, participants likely perceived larger uncertainty for the balloon’s size when they had chosen a smaller (2 or 3) than a larger number of pumps (9 or 10). Nevertheless, the number of pumps did not significantly predict counterfactual information seeking. These results are in contrast to previous findings that curiosity is evoked by uncertainty (van Lieshout *et al.* 2021). Instead of the number of pumps, we found significant effects of pump value; when the pump values were higher, participants were more risk-averse in indicating the number of pumps (Table S1—see online supplementary material) and more likely to seek the counterfactual information after gaining points (Table 1). These results suggest that participants’ behavior was guided by their goals to earn points throughout the task, and that their decision to seek counterfactual information may have been affected by this goal (e.g. a desire to check if their choice in the number of pumps was reasonable to earn points). It should be noted that the pattern was different for bust trials when the balloon exploded. In these trials, participants sought information significantly more when the number of pumps was larger than smaller (Table S3; Figure S5—See online supplementary material). Given that uncertainty for the balloon’s size should be higher when participants had chosen a larger (9 or 10) than smaller number of pumps (1 or 2) in these trials, it appears that the role of uncertainty is different, especially for counterfactual information seeking when the counterfactual outcome is better than the reality.

In addition, we found that counterfactual curiosity has downstream effects on behavior. Specifically, participants adjusted bidding behavior subsequently based on the missed opportunities, such that they chose to pump more after seeing larger missed opportunities (Table 3). Thus, even though the counterfactual information did not have utility value, the information still affected participants’ subsequent behavior. These behavioral effects were also accompanied by the activity in the caudate and NAcc; while none of our ROIs were predictive of changes in participants’ behavior in a subsequent trial, larger missed opportunities were associated with stronger activity in the caudata and NAcc. These results point to the likelihood that missed opportunities mediate the relation between striatal activity and subsequent changes in their behavior. Taken together, our results are in line with previous findings that the striatum is involved in the processing of regret and missed opportunities (Büchel *et al.* 2011, Brassen *et al.* 2012). Our results also suggest that the striatum is associated with people’s choice of behavior after regret (Nicolle *et al.* 2011).

Previous studies revealed that the dopaminergic reward systems are crucial not only for reward prediction error (i.e. the difference between an expected reward and an obtained reward) but also for counterfactual prediction error (i.e. the difference between an obtained reward and what could have been obtained; Kishida *et al.* 2016, Diederen and Fletcher 2021). Past studies also showed that the striatum is involved in the processing of regret and missed opportunities (Büchel *et al.* 2011, Brassen *et al.* 2012). Our results extend these findings by suggesting that the dopaminergic reward system is important in multiple ways in the processing of counterfactual information; it is involved not only in the processing value of counterfactual rewards but also in one’s decision to seek counterfactual information itself. At the same time, we did not find consistent activity patterns across our ROIs. For example, counterfactual information seeking was associated with the caudate and SN/VTA but not NAcc, whereas the size of missed opportunities was associated with the caudate and NAcc but not SN/VTA. However, these brain regions sometimes showed significant activity in our whole brain analyses (see Supplementary Materials). Thus, future research is needed to understand the specific role of each of the regions included in our analyses.

## Supplementary Material

nsag012_Supplementary_Data

## Data Availability

Behavioral data, codes to reproduce behavioral results, as well as analysis outputs are shared in the Open Science Framework (https://osf.io/w693x/overview). Unthresholded statistical maps are uploaded to the NeuroVault (https://neurovault.org/collections/WKDHXVZX/). Brain imaging data underlying this article will be shared on reasonable request to the corresponding author.
