# Supplementary material for: Role of the striatum in counterfactual information seeking
Source: Soc Cogn Affect Neurosci. 2026 Mar 10;21(1):nsag012. doi: 10.1093/scan/nsag012 (PMC13213454; doi:10.1093/scan/nsag012)
Supplement: nsag012_Supplementary_Data [file nsag012_supplementary_data.zip › 23-Mar-2026_035912_Rev2_SI_fin.docx]

**Supplemental Materials**

**Fig S1. Effects of outcomes on the choice to seek information about the balloon’s limit**

**Fig S2. Effects of waiting time cost and pump value on the choice to seek information about the balloon’s limit**

**Table S1. Effects of pump value on the number of pumps indicated during the bidding phase**

|  | **DV = Number of pumps** | | | | |
| --- | --- | --- | --- | --- | --- |
| *Predictors* | *Estimates* | *std. Error* | *df* | *t* | *p* |
| (Intercept) | 5.128 | 0.172 | 37 | 29.81 | < .001 |
| Pump value | -0.016 | 0.001 | 2901 | -12.20 | <.001 |
| **Random Effects** | | | | | |
| σ^2^ | 4.02 | | | | |
| τ_00_ _participant_ | 1.07 | | | | |
| ICC | 0.21 | | | | |
| N _participant_ | 38 | | | | |
| Observations | 2940 | | | | |

*Note.* The random slope effects for the pump value were not included due to the conversgence error.

**Table S2. Effects of experiences in previous trials on participants’ bidding choice in the following trial**

|  | **Across all trials** | | | | | |
| --- | --- | --- | --- | --- | --- | --- |
|  |  |  |  |  |  |  |
| *Predictors* | *Estimates* | *SE* | | *df* | *t* | *p* |
| (Intercept) | 4.3176 | | 0.20661 | 69.2455 | 20.897 | <.001 |
| Number of pumps in t-1 | 0.102 | | 0.01969 | 2784.371 | 5.179 | <.001 |
| Outcome in t-1  (bust = -1; bank =1) | 0.4632 | | 0.1297 | 30.20542 | 3.572 | <.001 |
| Choice in t-1  (seek = 1; no =-1) | -0.0327 | | 0.17636 | 35.97426 | -0.185 | .8539 |
| Outcome x choice interaction in t-1 | 0.0424 | | 0.19424 | 34.31377 | 0.218 | .8287 |
| **Random Effects** | | | | |  |  |
| σ^2^ | 4.17 | | | |  |  |
| τ_00_ _participant_ | 1.29 | | | |  |  |
| τ_11_ _participant.outcome t-1_ | 0.15 | | | |  |  |
| τ_11_ _participant.choice_t-1_ | 0.25 | | | |  |  |
| τ_11_ _participant.outcome x choice t-1_ | 0.28 | | | |  |  |
| ρ_01_ | -0.67 | | | |  |  |
|  | -0.57 | | | |  |  |
|  | 0.01 | | | |  |  |
| ICC | 0.19 | | | |  |  |
| N _participant_ | 38 | | | |  |  |
| Observations | 2808 | | | |  |  |

*Note.* The random slope effects for the number of pumps in t-1 were not included due to the convergence error.

**ROI Results during Choice Phase for the Effects of Pump Value and Cost**

Pump value was not significantly associated with our ROI’s activity for the bank trials during the choice phase (*p* = .26, .57, .56; Fig S3A). At the same time, there were still significant differences in the activity in the caudate and SN/VTA after controlling for the effects of pump value. Specifically, these regions showed significantly greater BOLD signals when participants decided to seek information than not to seek information (Fig S3B), *ts*(36) = 2.32, 2.38, *p*s = .039, .039 (FDR). Likewise, the magnitude of time cost was not significantly associated with our ROI’s activity for the bank trials during the choice phase (*p* = .75, .32, .52; Fig S3C). At the same time, the caudate and SN/VTA still showed significantly greater BOLD signals when participants decided to seek information than not to seek information after controlling for the effects of time cost (Fig S3D), *ts*(36) = 2.33, 2.37, *p*s = .039, .039 (FDR). We also multiplied the pump value and ballon size to estimate the expected value for each trial; but this expected value was not significantly associated with our ROIs activity during the choice phase (*p*s = .51, .53, .14, FDR; Fig S3E). In addition, even after controlling for the effects of expected value, the caudate and SN/VTA showed significant differences in their BOLD signals depending on the choice to seek counterfactual information, *ts*(36) = 2.45, 2.35, *p*s = .037, .037 (FDR; Fig S3F).

**Fig S3. Effects of pump values (A), time cost (C) and the expected value (E; pump value multiplied by the number of pumps) on activity in the bank trials during the choice phase. The effects of choice were still significant in the caudate and SN/VTA (B, D, F) after controlling for these effects.**

**
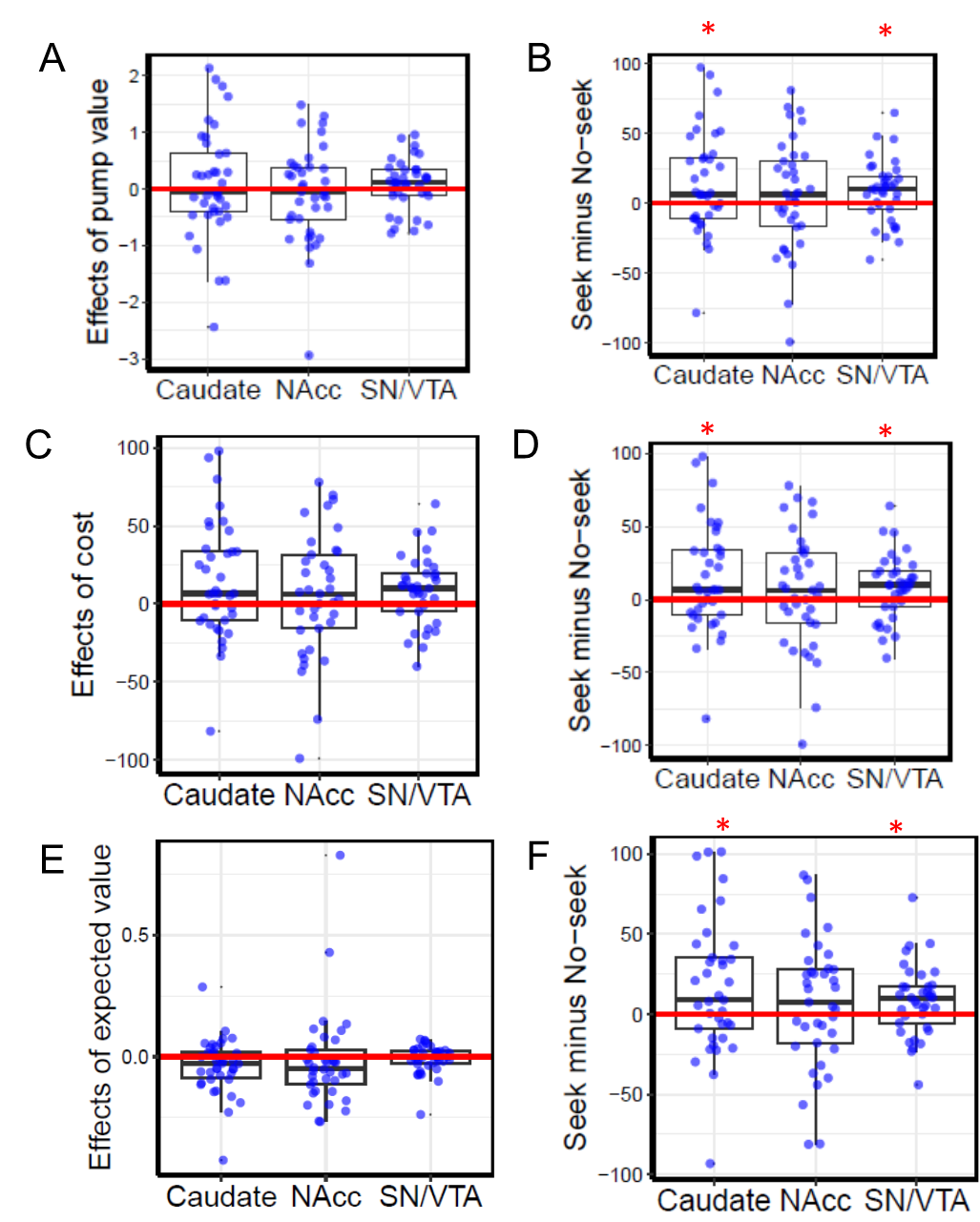
**

**Predictive Effects of Feedback- and Choice- Related Brain Activity**

BOLD signals in our ROIs were not significantly associated with the magnitude of changes in participants’ bidding choice — irrespective of whether we used signed changes (to represent an increase vs. a decrease in the number of pumps relative to a previous trial; *p*s > .60) or absolute changes (*p*s > 40; Fig S4). Likewise, BOLD signals in our ROIs during the choice phase were not significantly predictive of the magnitude of changes in their behavioral choice (*p*s > .80; Fig S5).

**Fig S4. Modulation effects of participants’ next bidding behavior on ROI activities during the feedback phase. None of the ROIs’ activity significantly predicted subsequent bidding behavior irrespective of (A) whether we focused on the signed changes or (B) absolute changes.**

**
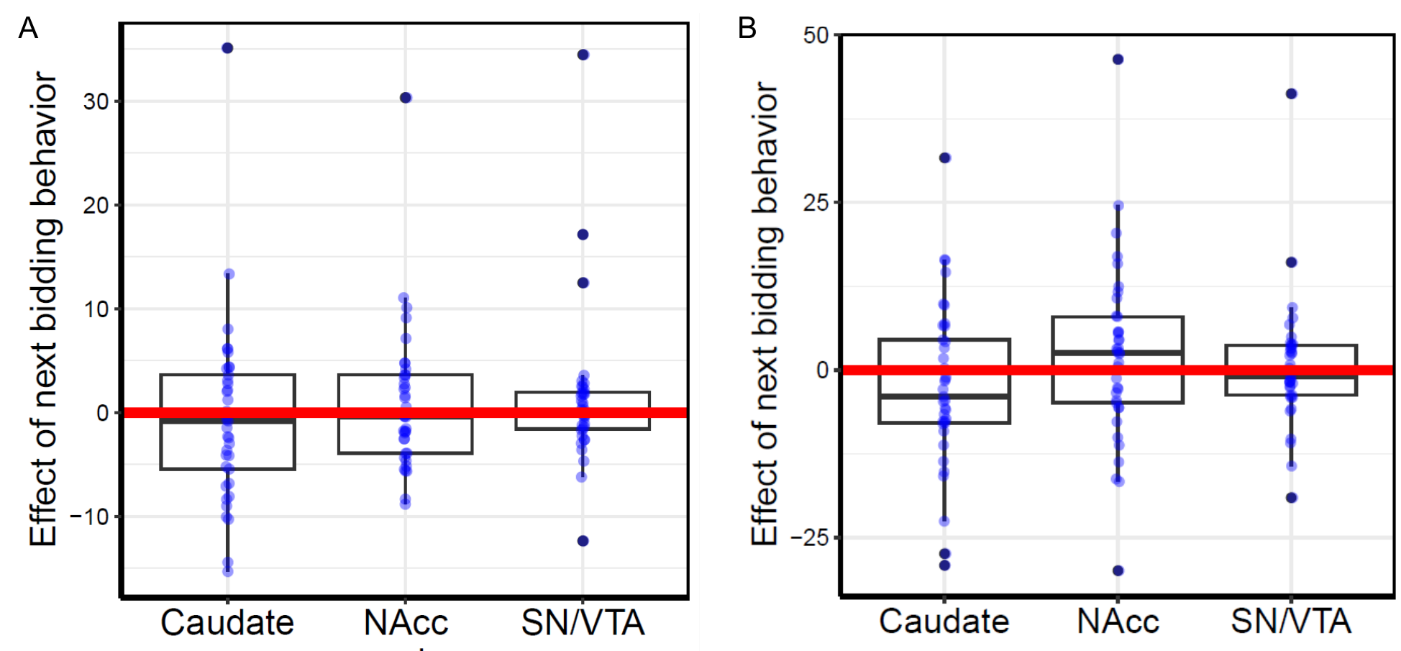
**

**Fig S5. Modulation effects of participants’ next bidding behavior on ROI activities during the choice phase (based on the signed changes).**

**Behavioral Results for Bust Trials**

Two participants did not seek information in the bust trials and one additional participant sought information only once in the bust trials; data from this participant thus did not have any variance in a difference score between the number of pumps indicated and the limit of the balloon and were not included when we analyzed the effects of the difference score in the bust trials.

In the bust trials, participants’ emotions at Time 2 did not significantly differ depending on whether they sought the information about the balloon’s limit (*M* = -.91, SD = 2.63) or not (*M* = -.24, SD = 1.40, *p* = .13). Thus, seeking information in the bust trials does not appear to induce strong negative emotions as observed in the bank trials. These results suggest that counterfactual curiosity has emotional costs primarily after gaining rewards.

In the main analysis on the bank trials, we examined the effects of missed opportunities using a difference score between the number of pumps participants indicated and the limit of the balloon for each trial. This difference score does not mean missed opportunities in the bust trials, where participants did not earn any points. Instead, it reflects the over-estimation of the balloon’s size. To examine the effects of the over-estimation, we ran a mixed effects modeling analysis and found a significant interaction between the choice and the over-estimation, *t*(1035.21) = -5.30, *p* < .001. Subsequent analyses revealed that when participants sought the information about the balloon’s limit, they reported more negative emotions at Time 2 when the over-estimation was smaller, *t*(30.43) = -2.51, *p* < .01. This is likely due to the fact that participants realized that they could have won the points if the size would have been slightly smaller. In contrast, when participants did not seek information, there were no significant effects of the over-estimation (*p* = .70).

We also examined the effects of pump value, time cost and the number of pumps in the choice to seek the limit of balloons in the bust trials (Table S3). As seen in the bank trials, participants chose to seek the limit of balloon when the time cost was lower. In addition, participants chose to seek information more when they chose a larger sized of the balloon during the bidding phase (Fig S5).

**Table S3. Effects of pump value, time costs and the number of pumps on counterfactual information seeking in the bust trials**

| *Predictors* | *Estimate* | *Std. Error* | *Z value* | *p* |
| --- | --- | --- | --- | --- |
| (Intercept) | -.596 | 0.24 | -2.517 | **0.012** |
| Pump value | 0.006 | 0.003 | 1.834 | 0.067 |
| Time cost | -0.580 | 0.064 | -8.952 | **<0.001** |
| Number of pumps | 0.087 | 0.039 | 2.188 | **0.029** |
| **Random Effects** | | | | |
| σ^2^ | 3.29 | | | |
| τ_00_ _participant_ | 1.87 | | | |
| τ_11_ _participant.number of pumps_ | 0.00 | | | |
| τ_11_ _participant.pump value_ | 0.00 | | | |
| ICC | 0.36 | | | |
| N _participant_ | 38 | | | |
| Observations | 1091 | | | |

*Note:* The random slopes for the cost were not included due to the convergence error.

**Fig S5. Effects of numer of pumps on counterfactual information seeking in the bust trials**

**ROI Analysis in the Bust Trials**

For bust trials, none of our ROIs showed significant effects during the choice phase depending on whether participants sought information for the ballon’s limit vs. not, *t*s(34) = .34, -.44, .09, *p*s = .92, .92, .92 (FDR), for the NAcc, caudate and SN/VTA respectively (Fig S6).

**Fig S6. The activity in our ROIs during the choice phase for bust trials.**

**Whole Brain Analysis**

We also performed a whole-brain analysis to examine how the choice to seek information affects BOLD signals beyond our ROIs during the choice and feedback phases. Outputs from the GLM analyses described above were entered into a random effects analysis using FSL’s FEAT FLAME 1. We employed cluster-based corrections for multiple comparisons with Gaussian random field theory (*Z* = 3.1; cluster significance: *p* = .05-corrected).

**Results from Whole Brain Analysis during Choice Phase (Bank Trials)**

None of our ROIs showed significant effects in the whole brain analysis, where we compared brain activities when participants chose to seek information vs. not during the choice phase. This is in contrast with the ROI results and suggest that the effects of choice on activity in our ROIs may have been relatively small in their effect size. However, the anterior cingulate (ACC) and the orbitofrontal cortex/insular showed greater activity when participants decided to seek information than when they decided not to in the bank trials (Fig. S7A). The reversed contrast showed significant clusters only in the occipital area (Fig S7B).

**Fig S7. Whole brain results during the choice phase for bank trial**


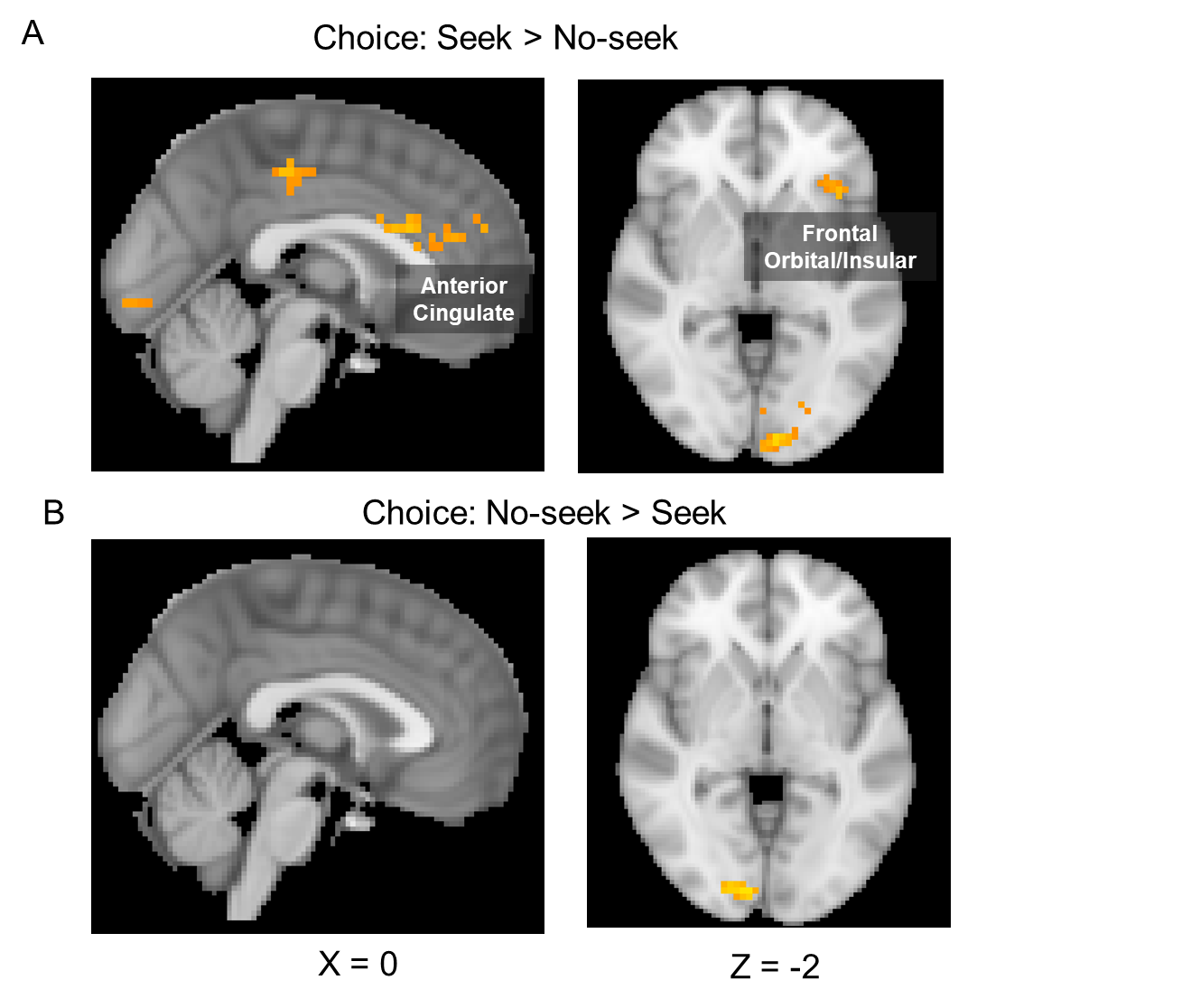


**Results from Whole Brain Analysis during Feedback Phase (Bank Trials)**

For the feedback phase, the left caudate, the putamen, and the brainstem showed significantly greater activity when participants chose to seek information vs. not (Fig S8). The brainstem also showed greater activity after seeking information than not; the cluster overlapped with the SN/VTA mask (Fig S8). These results are consistent with our prediction and prior findings that the dopaminergic regions in the brain are relevant to counterfactual information and resulting regret. It should be noted that these results are in contrast to results from our ROI analyses that did not show significant effects in these regions. In our ROI analyses, we simply examined the average BOLD signals across all voxels in these brain regions; in contrast, we examined each voxel separately in the whole brain analysis; examining each voxel may have helped us to identify voxels that are particularly sensitive to counterfactual information. In addition, the contrast showed a wide-spread cluster across the bilaterial thalamus, superior frontal gyrus, and frontal orbital cortex/insular. The reversed contrast showed significant activity in the medial prefrontal cortex and precuneus — regions typically called the default mode network.


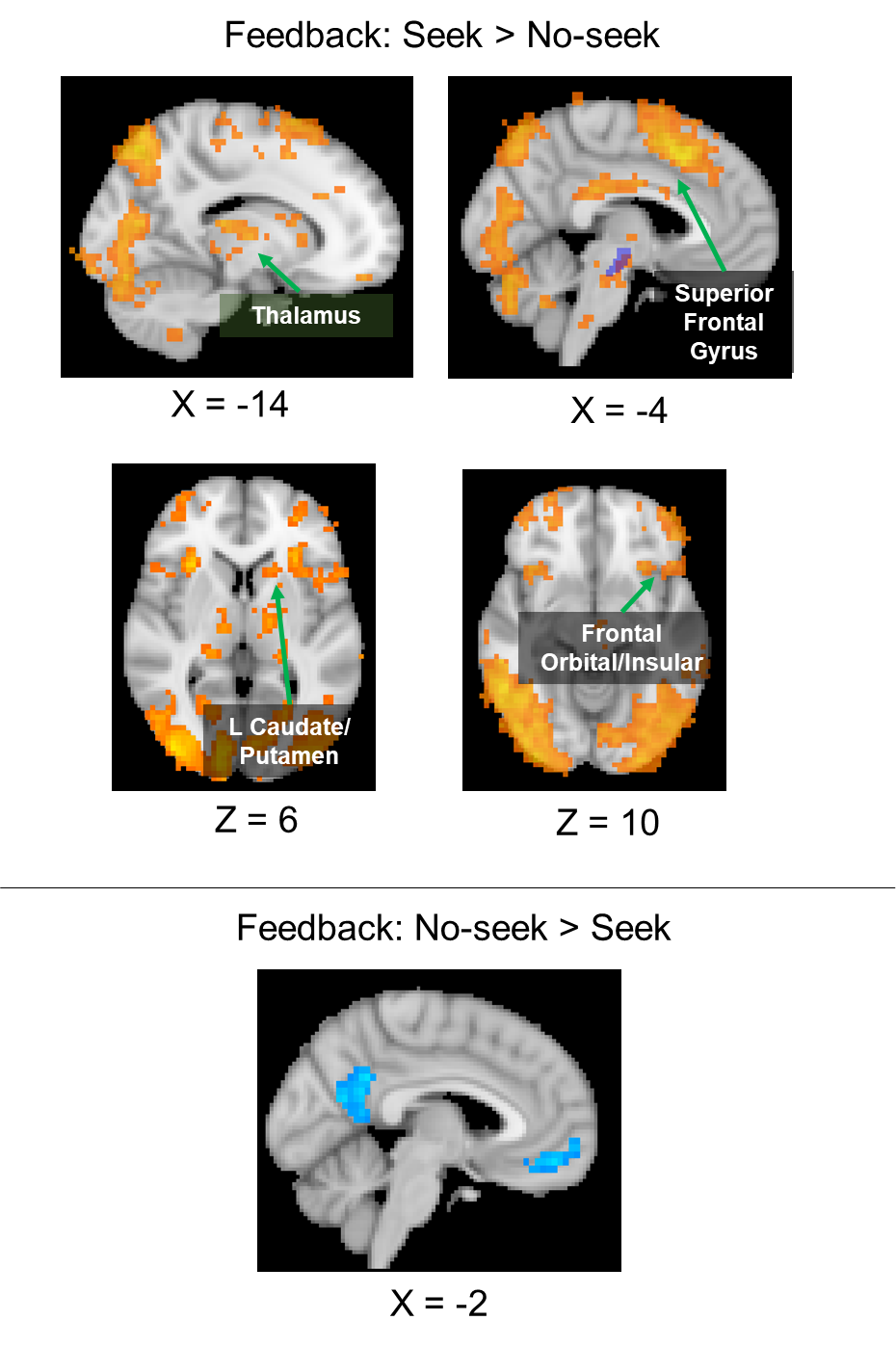


**Fig S8. Whole brain results during the feedback phase for bank trial**

We next performed another whole brain analysis with parametric modulation to investigate the effects of missed opportunity. This analysis confirmed the results from the ROI analysis that the caudate and the NAcc showed significantly greater activity when the missed opportunities were larger after seeking information in the bank condition during the feedback phase (Fig S9).


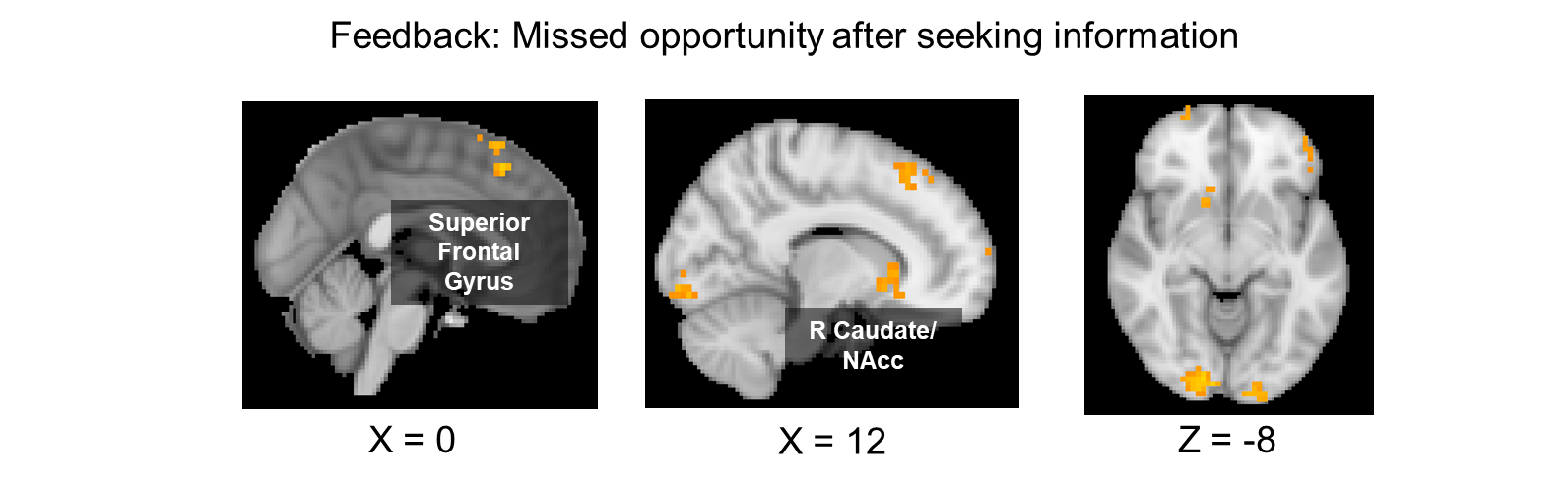


**Fig S9. Whole brain results for the effects of missed opportunity during the feedback phase for bank trial**

**Whole Brain Analysis during Choice Phase (Bust Trials)**

When we compared brain activities when participants chose to seek information vs. not durng the choice phase in the bust trials, there was a significant cluster only in the occipital cortex. The reversed contrast also showed a significant cluster in the occipital cortex (Fig S10).


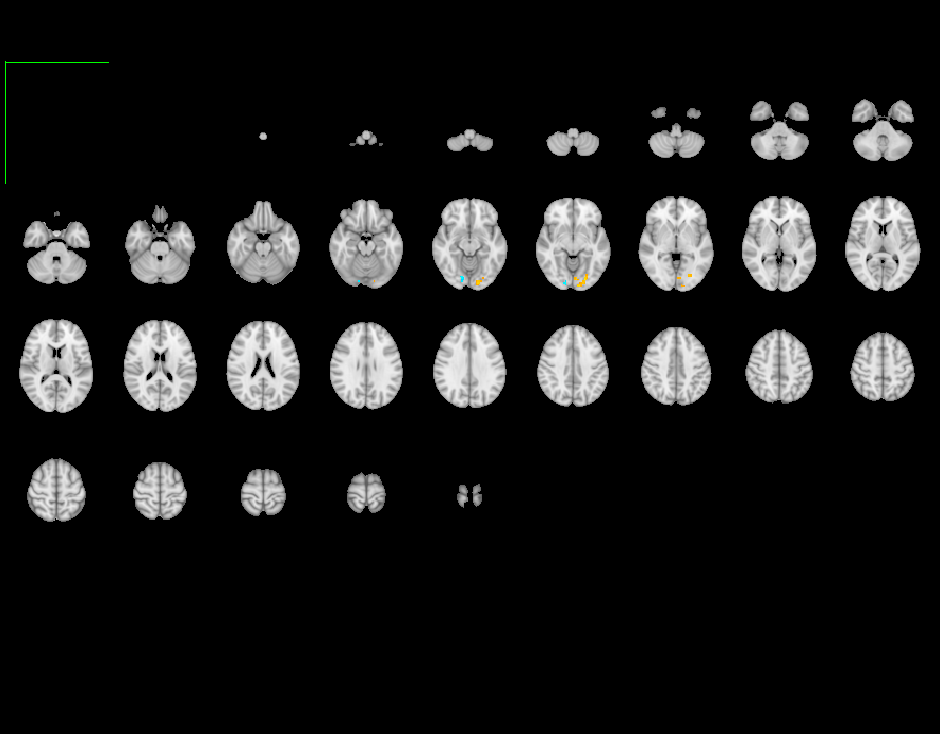


**Fig S10. Whole brain results for the choice phase. Yellow areas: Seek > No-seek. Blue areas: No-seek > Seek.**

**Whole Brain Analysis during Feedback Phase in Bust trials**

As observed in bank trials, there were wide-spread activities across the left thalamus, bilateral superior frontal gyrus, bilateral frontal orbital cortex/insular when participants chose to seek information vs. not (Fig S10). However, unlike the results from the bank trials, there were no significant clusters in the striatum nor in the brain stem for the contrast as well as the reversed contrast (Fig S11). There were no significant effects for the over-estimation either.


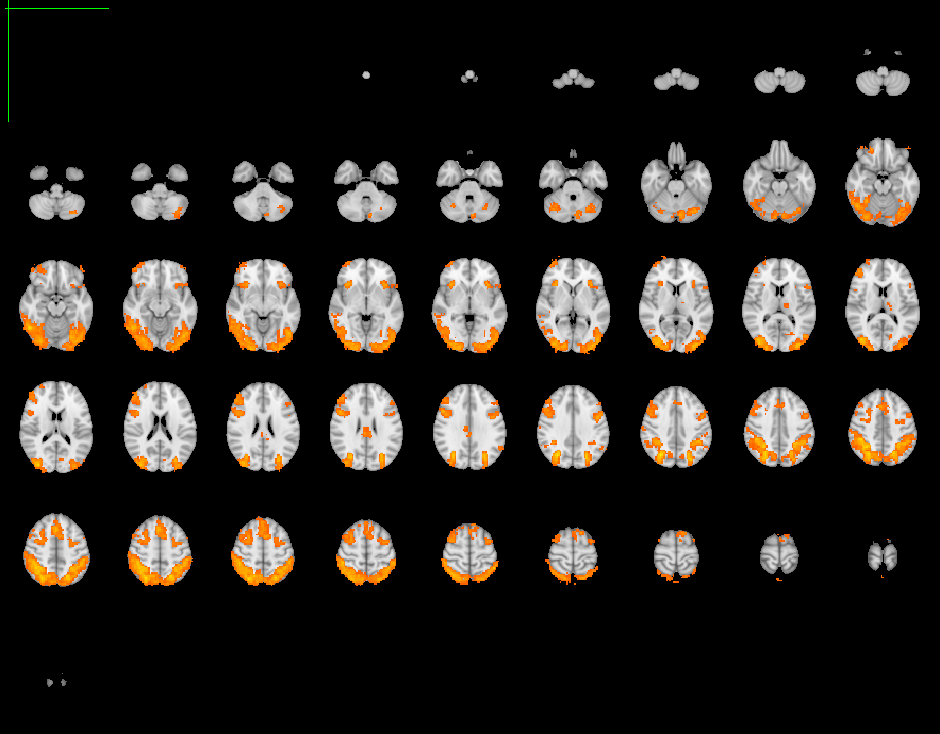


**Fig S10. Whole brain results for the feedback phase (seek > no-seek in the bust trials).**


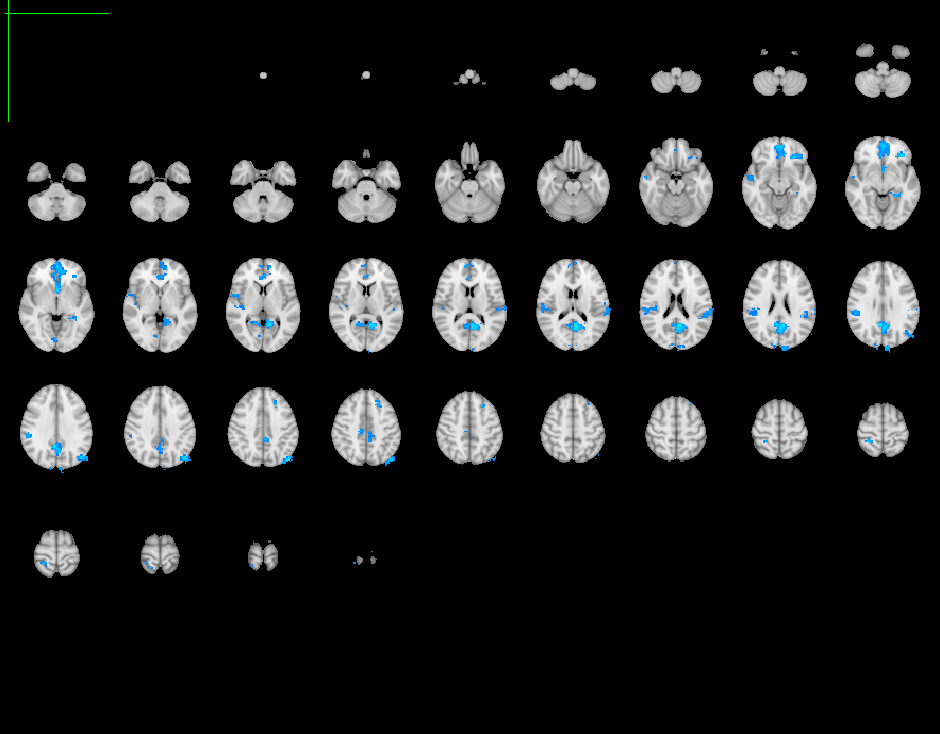


**Fig S11. Whole brain results for the feedback phase (no-seek > seek in the bust trials).**
